# The epidemiology of homicide perpetration by children

**DOI:** 10.1186/s40621-017-0102-2

**Published:** 2017-02-13

**Authors:** David Hemenway, Sara J. Solnick

**Affiliations:** 1 Harvard TH Chan School of Public Health, 677 Huntington Avenue, Boston, MA 02115 USA; 2 University of Vermont, 94 University Place, 237 Old Mill, Burlington, VT 05405 USA

**Keywords:** Children, Homicide, Perpetrator, Guns

## Abstract

**Background:**

The United States has by far the highest rates of homicide perpetration among high-income countries. The perpetration of homicide by children is often newsworthy, but little is known about the incidence or the circumstances of child homicide perpetration.

**Methods:**

We use data from the sixteen states reporting to the National Violent Death Reporting System (NVDRS) for all years 2005–2012. We read every violent death report that was classified a homicide with a child suspect (aged 0–14). To help ensure that we did not miss any homicide cases we also read those classified as an other-inflicted unintentional firearm injury death with a child shooter, to determine if they were actually homicides.

**Results:**

There were 154 child suspects, which corresponds to an average annual rate of 1.2 child perpetrators per million child population. We estimate for the United States as a whole, 74 children per year were homicide perpetrators. Nearly 90% were boys, 79% were aged 13–14, and another 13% were aged 11–12. We created five categories, which accounted for over 70% of events with sufficient information to determine what happened: (1) The caretaker, a juvenile, typically an older brother, is given the responsibility to care for an infant. The homicide usually occurs in a residence, and blunt force is used (no guns); (2) Killing an adult family member, typically a parent or grandparent. These cases usually occur in a residence, and the child uses either a gun or a knife; (3) Impulsive shooting during play, the child typically shoots a sibling or friend. Except for some notion of momentary anger, these cases look much like unintentional firearm fatalities; (4) Robbery, a group of youth are trying to steal money, usually from an adult; and (5) Group Assault, a group of youth are fighting, usually other youth.

**Conclusions:**

Child homicide perpetrators are typically boys who use guns, and the events can be classified into a small number of relevant categories. Such a categorization of events is useful for understanding the problem and determining solutions.

## Background

The United States has by far the highest rates of homicide perpetration and victimization among high-income countries (Grinshteyn and Hemenway 2016). The perpetration of homicide by children sometimes makes the evening news. Information that has not previously been available to reporters is how common it is for children to murder, and in what circumstances. In this paper we use data from the National Violent Death Reporting System (NVDRS) to help determine the epidemiology of child (ages 0–14) homicide perpetration and to create a typology of events.

A number of books have been written about “children who kill” which typically examine a dozen or so cases, where many of the child killers are older adolescents (Davis 2004; Ewing 1990). There also have been many case studies examining the psychiatry and environments of child perpetrators of homicide (Shumaker and Prinz 2000).

A small literature also exists that uses crime databases [e.g., the Supplementary Homicide Reports (SHR), the National Incident-Based Reporting System (NIBRS)] to focus on a variety of related issues, including family violence by perpetrators of any age such as offspring killing parents (Fegadel and Heide KM. Offspring-perpetrated familicide: examining family homicides involving parents as victims. Int J Offender Ther Comp Criminol 2017; Heide and Petee 2007), murder by brothers (Walsh and Krienert 2014), sibling homicide (Underwood and Patch 1999), patricide and steppatricide (Heide 2014), and matricide and stepmatricide (Heide 2013).

Most relevant to our paper are studies using criminal databases that have looked at such topics as girl homicide perpetrators (Heide and Sellers 2014) and male and female children who murder (Sellers and Heide 2012). These articles compared younger girls and older girl homicide perpetrators, and boy versus girl perpetrators.

As far as we can tell, no prior study has used NVDRS data to examine homicide perpetration by children. The SHR has data problems (Pizarro and Zeoli 2013) including the non-reporting of homicides, and missing and inaccurate reporting regarding victim-offender relationship and circumstances. Such data problems are less serious in the NVDRS, which combines multiple databases, and incorporates SHR data. The NVDRS also has the advantage of providing narratives about the event from both the police and the medical examiner. By reading these narratives we were able to create a typology of child homicide events that we believe will be helpful for prevention efforts.

## Methods

We use data on child perpetrators of homicide from states reporting to the National Violent Death Reporting System (NVDRS) for all years 2005–2012. Sixteen of the reporting states met that criteria (Alaska, Colorado, Georgia, Kentucky, Maryland, Massachusetts, New Jersey, New Mexico, North Carolina, Oklahoma, Oregon, Rhode Island, South Carolina, Utah, Virginia, and Wisconsin). Ohio only began providing data in 2010 and was excluded.

The NVDRS is a state-based surveillance system that links data from the death certificate, law enforcement records, and coroner/medical examiner records on deaths due to suicide, homicide, legal intervention, unintentional firearm injury, and firearm fatalities of unknown intent (Paulozzi et al. 2004; Hemenway et al. 2009; Barber et al. 2013). The records are incident-based and include information on the persons (victims and perpetrators), weapons, and circumstances involved. In addition to the coded data, the abstractor writes two incident narratives summarizing the findings from the coroner/medical examiner records and from law enforcement records. Data are collected and linked at the state level, stripped of personal identifiers, and forwarded to the Centers for Disease Control and Prevention (CDC). Data for this study come for the NVDRS Restricted Access Dataset (RAD). The CDC data use agreement for RAD forbids presenting cell sizes with fewer than 10 individuals. This study was deemed exempt by the Harvard Human Subjects Institutional Review Board since it uses only data which are publically available to bona fide researchers.

The data do not tell us whether or not the child eventually was found legally responsible as a murderer; we examine all cases in which the child was a suspected perpetrator of the homicide at the time the NVDRS information was recorded. The authors of this paper read every violent death report that was classified as a homicide with a child (aged 0–14) suspect. Two cases that had been classified as homicide were suicides according to the narratives and we re-classified them accordingly. Twenty other cases were moved from homicides to unintentional gun deaths. The NVDRS has been shown to sometimes incorrectly classify cases between unintentional firearm deaths and child firearm homicides (Barber and Hemenway 2011). To help ensure that we did not miss any cases we also read those classified as other-inflicted unintentional firearm injury death with a child shooter. None of these appeared to be a homicide. The narratives were read by both authors, and in the very few cases of disagreement about whether or not this was a child perpetrated homicide, we met and reached agreement.

A priori, we divided the perpetrator age groups into ages 0–10, 11–12 (pre-teen) and 13–14 (early teen). For simplicity of exposition, when we report on incidents in the tables and there was more than one child suspect, we report the age of the youngest suspect. Number of child years exposure is defined as the sum of the population of children living in the sixteen NVDRS states each of the eight years.

We created the categories for these homicides inductively, informed by our background in injury prevention. As there was no established categorization for this particular age group of perpetrators, we read all the cases with an open mind. Five major types of cases emerged. There were no disagreements about the classification of individual cases.

## Results

There were 146 incidents and 151 deaths (in 5 incidents two people were killed) with a homicide perpetrator aged 0–14 in the 16 states for the eight-year period (Tables 1 and 2). In 94 of the 146 incidents there was one suspect; in 52 there were multiple suspects. In eight of the multiple suspect cases the additional suspect was also aged 0–14. Thus in the 146 incidents, there were 154 child suspects.

**Table 1:** Characteristics of children (0–14) as suspects in homicide

Age of Child Suspect	# Child Suspects	Total Child-Years (000)	Homicides/ 1 Million Child-Years	% Male Suspect	% Black
0–10	12	94,274	0.1	92%	54%
11–12	20	17,464	1.1	85%	64%
13–14	122	17,623	6.9	88%	63%
All	154	129,361	1.2	88%	62%

**Table 2:** Characteristics of events by age of youngest suspect

Age of Youngest Suspect	# Events	# Victims	% Firearm	% of Firearms that are Handguns	Age of Victim		
					% 0–14	% 15–19	% 20+
0–10	12	12	58%	17%	50%	8%	42%
11–12	20	22	59%	62%	55%	5%	41%
13–14	114	117	61%	75%	18%	20%	62%
All	146	151	61%	68%	26%	17%	57%

We estimate that this represents some 1.2 children as homicide perpetrators per million child years. The child population of the 16 states is 26% of the total US child population. In addition, Vital Statistics data for the years 2005–2012 show that the number of total firearm deaths of children in these 16 states represents 26% of the total firearm deaths for the US child population. Therefore, extrapolating the rate to the entire population, we estimate that the total number of children perpetrating homicide in this eight-year period was 154/.26 = 592, or approximately 74 per year.

Close to nine out of ten (88%) of the perpetrators were boys (Table 1). Over three quarters (79%) were aged 13–14, and another 13% were aged 11–12. Perpetrators were disproportionately Black (62% of suspects, while Black children make up 15% of the US child population). Guns were used in 61% of the events, and two-thirds of these guns were handguns (Table 2).

After reading the cases we created five main categories of child perpetrated homicides, which account for 71% of all incidents, and over three quarters of the cases in which there was sufficient information to determine what happened (in ten cases there was little or no information about the circumstances of the event from either the police or medical examiner reports). In the first three categories the victim is a family member or close friend; in the latter two categories the victim is an acquaintance or stranger (Table 3).

**Table 3:** Characteristics by type of incident

	Incident Type					
	Suspect was Caretaker	Family Member	Impulsive Shooting during Play	Robbery	Group Assault	Other
Number of incidents	13	23	20	21	26	43
Number of victims	13	25	20	23	26	44
Number of child suspects	13	24	21	23	28	45
Incidents with only one suspect	100%	84%	80%	26%	15%	84%
Age of Youngest Suspect						
%13–14	54%	77%	52%	100%	82%	85%
Sex of Youngest Suspect						
% Male	77%	81%	95%	96%	86%	91%
Weapon						
% Firearm	0%	52%	100%	70%	68%	57%
% Firearms are handguns	--	44%	37%	82%	83%	86%
Age of Victim						
% 0–4	92%	0%	5%	0%	0%	0%
% 5–12	8%	8%	35%	0%	0%	11%
% 13–19	0%	0%	50%	13%	35%	34%
% 20+	0%	92%	10%	87%	65%	55%

Category 1 (13 cases): The Caretaker: The child is given the responsibility of being the caretaker for a young child--92% of these cases involve the killing of a 0–2 year-old-- and the young relative (often the brother) inflicts severe trauma on that infant, in the residence. Guns are not involved.

Category 2 (25 cases) Killing a Family Member: The perpetrator kills an adult (92% of cases) family member, almost always a parent or grandparent, and virtually always in the residence. Guns are used about half the time.

Category 3 (20 cases) Impulsive Shooting During Play: The perpetrator shoots someone, typically a non-adult (90% of cases the victim is aged 0–19) in what is very similar to an unintentional shooting, except for immediate intent. The victim is usually a sibling or peer friend, and the event often occurs during an argument, though some of the cases are unclear. Often the suspect says he did not believe the gun was loaded. The majority of these guns are long guns.

Category 4 (23 cases) Robbery: Usually a group (74% of time) of juveniles, including the suspect, is trying to steal money from the victim, typically an adult (87% of cases).

Category 5 (26 cases) Group Assault: A group of juveniles (often a gang) fights with or simply shoots another individual or group. All victims are teenagers or older. In the large majority of cases (84%) more than one individual is considered a suspect.

In both Robberies and Group Assaults, guns are used two-thirds of the time and over 80% of the guns used are handguns.

Of the 146 events, compared to the child population, these were disproportionately likely to occur in large urban areas (29% of events, 15% of the child population) and less likely to occur in the suburbs (23% of events, 37% of the child population). Small urban areas had 31% of events and 32% of the child population while rural areas had 17% of events and 16% of the child population (not shown).

## Discussion

We estimate that there are approximately 74 children (ages 0–14) who murder someone each year in the United States. This may be a conservative estimate, since some unsolved homicides may have child perpetrators. In almost all of the known cases, only one person is killed. In our dataset, guns were used in over 60% of the deaths, and in more than ¾ of all the deaths, the perpetrator was 13–14 years old.

These results are consistent with national data from the Supplementary Homicide Reports (SHR). For the eight years 2005–2012, the SHR reports somewhat under 100 juvenile homicide offenders aged 0–14; 65% were aged 14 (http://www.ojjdp.gov/ojstatbb/offenders/qa03104.asp?qaDate=2014&text=yes). Children aged 0–14 represent less than 1% of all homicide perpetrators in the United States, many of these homicides appear to be preventable, and these killings are tragedies, not only to the victim but to the child perpetrators. Also consistent with the SHR is the disproportionate number of Black perpetrators.

While few academic studies examine the exact age group that we do, or look at all murders rather than particular types of murder perpetrated by children (e.g., sibling homicide), our results appear generally consistent with the literature (Shumaker and Prinz 2000; Fegadel and Heide 2017; Heide and Petee 2007; Walsh and Krienert 2014; Underwood and Patch 1999; Heide 2014; Heide 2013; Heide and Sellers 2014; Sellers and Heide 2012). For example, boys are far more likely than girls to be the homicide perpetrator and older children are far more likely than younger children to kill an adult.

After reading all the cases we created five main categories of child homicide perpetration. Other authors--who have typically focused more on older adolescents-- have created different categories. For example, one divided killings by youth into four categories: family, theft-related, sexual, and “crazy killings” (Ewing 1990). Another way adolescent offenders have been categorized is one based on the circumstances of the event: (a) psychotic (youth with severe mental illness); (b) conflict (youth engages in an argument when the killing occurred); and (c) crime (youth who killed during the commission of another felony (Cornell et al. 1987)). We focus entirely perpetrators aged 0–14 and create what we hope are more appropriate categories for this age group, categories which are useful for prevention.

Our categories are (1) the caretaker, where a juvenile is put in charge on a baby, and kills it typically with blunt force. The death occurs in a residence. As with most infant homicides (Fujiwara et al. 2009), guns are rarely if ever used. A second category (2) is the killing of an adult family member, typically a parent or grandparent. Like the first category, almost all these events occur in a residence. The child perpetrator typically uses a weapon, either a gun or knife.

The third category, (3) impulsive shooting during play, looks similar to an unintentional firearm fatality (Hemenway and Solnick 2015). The victim is either a relative (sibling) or a close friend and is shot. Virtually every one of these killings could probably have been prevented had the child not had easy access to a firearm.

The fourth (4) and fifth (5) categories involve another crime, either a robbery or assault. The perpetrator is rarely alone, but is working with other teenagers. The assaults often involve inter-gang rivalries.

To effectively prevent a problem usually requires understanding what the problem is. Categorizing child homicide into main categories can help direct prevention efforts. For example, a way to reduce Category 1 homicides (the caretaker) is not to put children in complete charge of infants. A way to reduce Category 3 homicides (impulsive shooting during play) is to limit easy access to firearm for 10–14 year olds. Categories 4 and 5 illustrate the importance of groups of teenagers creating problems and committing crimes. Prevention efforts may be wise to focus not so much on individuals, but more on groups.

In over half of homicides perpetrated by children, the child used a gun. The percentage rises to over 60% when we exclude very young victims (e.g., category 1) (not shown). The gun makes it possible for children to use deadly force. Many child perpetrated homicides could probably be prevented if children did not have easy access to firearms.

This study has various limitations. First, we provide data only for 16 states for eight years. However, while the states were not randomly selected, they appear to be fairly representative of the United States, including states that are urban and rural, east and west coast, northern and southern, high gun prevalence and low gun prevalence. Compared to children in the entire US, children in these sixteen states are equally likely to be native born, live in a home with married parents, and not live in poverty. Children in the NVDRS states are more likely to be non-hispanic white (58% vs 54%) or non-hispanic black (17% vs 14%) and less likely to be Hispanic or Latino (16% vs 23%). They are equally likely to be Other race (9%) (not shown). Second, not all cases had a narrative, and such cases had to go into the “other” category. Even among the cases with narratives, almost one quarter did not fit neatly into one of our five categories. Third, in some homicides there is no suspect, so some cases with a child perpetrator may not have made it into our list of cases. In addition, suspects may not always be the actual perpetrators, though we treat them as such in this paper. Fourth, there were only 146 events. This small sample size further limits the confidence concerning how closely any point estimates drawn from the data correspond to the true values (e.g., the percentage of homicides perpetrated by children in which a parent or guardian is killed). Fifth, while we believe the categories of cases we developed can be useful, other researchers might read the narratives and create other classifications.

Finally, there is much information about the perpetrator, the victim, and the circumstances of the event that are not collected routinely in the NVDRS, or its four component data sources (i.e., Vital Statistics, Supplementary Homicide Reports, medical examiner/coroner reports, crime lab data). For example, there is little to no information about a long list of risk factors for violence such as prior gun carrying (Dodson 2016), hyperactivity or drug use, poor parenting, income inequality (World Health Organization 2015) or modifiable neighborhood features such as street lighting and unmaintained vacant lots (Culyba et al. 2016). Simple demographic information is also generally lacking, such as education or household income of the perpetrator or victim (or their parents). Though useful for researchers, such data are either not readily available to the individuals filling out the reports, or the abstractors, or are not deemed important enough to collect given the mission of the agencies involved.

## Conclusions

We estimate that approximately 74 children aged 0–14 murder someone each year. About nine out of ten of these children are boys, some 60% use firearms, close to 80% are 13 or 14 years of age, and most victims are adults. However, these broad generalities mask very different types of homicide. For example, for one of our categories of cases—the child caretaker—the suspects never use firearms and the victims are almost always infants under 2 years of age. In another category, impulsive shooting during play, the event is quite similar to an unintentional shooting, the suspect always uses a gun and the age of the victim is typically within a few years of the shooter. In the three of our categories of cases the perpetrator typically acts alone; in two (robbery and group assault), the perpetrator is part of a group.

We believe that effective prevention requires an understanding of the circumstances of the event, which is why creating useful categories is so important. It needs also to be emphasized that preventing a homicide by a child not only saves the victim, but it may also “save” the life (improve the life chances) of the child perpetrator.

## Abbreviations

CDC: Centers for disease control and prevention
NIBRS: National incident-based reporting system
NVDRS: National violent death reporting system
RAD: Restricted access dataset
SHR: Supplemental homicide reports
